# Biobehavioral Assessments in BACPAC: Recommendations, Rationale, and Methods

**DOI:** 10.1093/pm/pnac175

**Published:** 2022-11-12

**Authors:** Carol M Greco, Ajay D Wasan, Michael J Schneider, Wolf Mehling, David A Williams, Jessa Darwin, Steven E Harte

**Affiliations:** Department of Psychiatry, University of Pittsburgh School of Medicine, Pittsburgh, Pennsylvania, USA; Department of Physical Therapy, School of Health and Rehabilitation Science, University of Pittsburgh, Pittsburgh, Pennsylvania, USA; Department of Anesthesiology and Perioperative Medicine, School of Medicine, University of Pittsburgh, Pittsburgh, Pennsylvania, USA; Department of Physical Therapy, School of Health and Rehabilitation Science, University of Pittsburgh, Pittsburgh, Pennsylvania, USA; Clinical and Translational Science Institute, University of Pittsburgh, Pittsburgh, Pennsylvania, USA; Department of Family and Community Medicine, University of California San Francisco, San Francisco, California, USA; Chronic Pain and Fatigue Research Center, Department of Anesthesiology, University of Michigan Medical School, Ann Arbor, Michigan, USA; Department of Psychiatry, University of Michigan Medical School, Ann Arbor, Michigan, USA; Department of Internal Medicine-Rheumatology, University of Michigan Medical School, Ann Arbor, Michigan, USA; Department of Physical Medicine and Rehabilitation, University of Pittsburgh School of Medicine, Pittsburgh, Pennsylvania, USA; Chronic Pain and Fatigue Research Center, Department of Anesthesiology, University of Michigan Medical School, Ann Arbor, Michigan, USA; Department of Internal Medicine-Rheumatology, University of Michigan Medical School, Ann Arbor, Michigan, USA

**Keywords:** Behavioral Assessments, Psychosocial Assessments, Patient-Reported Outcomes, Quantitative Sensory Testing, Chronic Low Back Pain

## Abstract

The Biobehavioral Working Group of BACPAC was charged to evaluate a range of psychosocial, psychophysical, and behavioral domains relevant to chronic low back pain, and recommend specific assessment tools and procedures to harmonize biobehavioral data collection across the consortium. Primary references and sources for measure selection were the Initiative on Methods, Measurement, and Pain Assessment in Clinical Trials, the Minimum Data Set from the National Institutes of Health (NIH) Research Task Force on Standards for Chronic Low Back Pain, the Patient-Reported Outcomes Measurement Information System, and NeuroQOL. The questionnaire’s recommendations supplemented the NIH HEAL Common Data Elements and BACPAC Minimum Data Set. Five domains were identified for inclusion: Pain Characteristics and Qualities; Pain-Related Psychosocial/Behavioral Factors; General Psychosocial Factors; Lifestyle Choices; and Social Determinants of Health/Social Factors. The Working Group identified best practices for required and optional Quantitative Sensory Testing of psychophysical pain processing for use in BACPAC projects.

## Introduction

Chronic low back pain (cLBP) currently ranks as the most disabling and costly condition affecting the US population [1]. Efforts to understand and treat cLBP have historically focused on biological mechanisms along with biomedical and biomechanical treatments. In recent decades, the biopsychosocial model of pain has broadened our understanding of the many factors that are associated with pain processing in the context of cLBP [2]. Greater understanding of the interplay between biological, psychological and social factors in the production of pain facilitates better precision in matching treatments with the needs of patients [3]. Examples of factors with known influence on pain processing include emotional state, cognitive or thinking styles, habitual coping methods, behavioral lifestyle choices, socioeconomic factors, history of trauma and memories of past pain experiences, and availability of social supports.

This article describes the methods and recommendations of the BACPAC Biobehavioral Research Working Group (BWG). The charge and responsibilities of the BWG were to examine a range of psychosocial, psychophysical, and behavioral domains relevant to the various BACPAC intervention and phenotyping projects, to make recommendations for harmonizing the domains to be assessed, and to recommend specific assessment tools and procedures.

Deliverables of the BWG included developing a set of recommendations regarding patient-reported outcomes (PROs)/psychosocial domains and specific questionnaires to 1) assess meaningful outcomes following interventions (outcomes assessment) and 2) contribute to prediction of who would respond to which specific interventions (phenotyping for precision medicine). The recommendations of a broader set of questionnaires to be included in BACPAC projects were meant to supplement the National Institutes of Health (NIH) HEAL Common Data Elements (CDE) and the BACPAC Minimum Dataset (BMD) of PROs and demographics. The BWG also provided best practice guidelines for quantitative sensory testing (QST) in BACPAC projects [4]. Table 1 provides a list of abbreviations. Although harmonization across sites was an important goal, we recognized that testing sites would choose the measures and procedures most pertinent to their projects and would strongly consider participant burden. The overall goal was to find a balance between maximizing harmonization for the assessment tools between the various BACPAC sites and allowing flexibility for individual teams’ objectives.

**Table 1.** Abbreviations

BACPAC	Back Pain Consortium (BACPAC) Research Program
BMD	BACPAC minimum dataset
BWG	Biobehavioral WG
CDE	Common data elements
cLBP	Chronic low back pain
CPM	Conditioned Pain Modulation
HEAL	The Helping to End Addiction Long-term^SM^ Initiative
IMMPACT	Initiative on Methods, Measurement, and Pain Assessment in Clinical Trials
MDS	Minimum dataset
MRC	Interdisciplinary Mechanistic Research Center
NIH	National Institutes of Health
NIMHD	National Institute on Minority Health and Health Disparities
PPT	Pressure Pain Threshold
PROMIS	Patient-Reported Outcomes Measurement Information System
PROs	Patient-Reported Outcomes
QOL	Quality of life
QST	Quantitative Sensory Testing
SDoH	Social determinants of health
WG	Working Group

* Abbreviations for questionnaires are included in Table 2.

## Methods

### BWG Membership

The BWG was composed of self-nominated investigators from the BACPAC sites, including the directors of the Behavioral Cores at each BACPAC Mechanistic Research Center (MRC). The BWG members were behavioral scientists and physicians with expertise in PROs, psychosocial assessment, clinical pain assessment and treatment, and psychophysical assessment using quantitative sensory testing. Of note, several members of the BWG served on additional BACPAC WGs, including the Minimum Data Set WG, Theoretical Model WG, and Biomechanical and Physical Function WG. This overlap allowed for greater integration and harmonization across projects and fostered ongoing connections for potential new collaborative research endeavors.

### BWG Procedures

The BWG established a schedule of online meetings. In initial discussions about its charge and deliverables, members of the BWG self-selected into two subgroups: 1) PROs/Psychosocial Questionnaires and 2) Psychophysical Assessment/QST.

The BWG PROs/Psychosocial Questionnaires subgroup used a literature review, discussion, and consensus decision-making approach to identify important assessment domains and concepts, and to recommend specific questionnaires for inclusion in BACPAC projects. The BWG PROs/psychosocial questionnaires members reviewed existing publications regarding psychosocial, behavioral, and pain-related domains. An iterative process identified domains based on their contribution to understanding the cLBP patients’ experience and on their prognostic value for treatment outcomes. Primary references were the Initiative on Methods, Measurement, and Pain Assessment in Clinical Trials (IMMPACT) recommendations [5–11] and the NIH Research Task Force Minimum Data Set (MDS) [12]. We also evaluated NeuroQOL and Patient-Reported Outcomes Measurement Information System^®^ (PROMIS) measures [13]. Advantages of PROMIS questionnaires include their rigorous instrument development methodology [14], simple score interpretation with mean T-score of 50 and Standard deviation of 10, and the availability of comparison tables so that PROMIS scores can be “linked” or translated into scores on other frequently used or “legacy” questionnaires [15–17]. The BWG also considered the domains and conceptual areas identified during the comprehensive literature reviews conducted by the Theoretical Models WG. Once conceptually important domains were identified, we evaluated specific questionnaires for inclusion in the list of recommended measures. We evaluated the evidence for their validity as prognostic indicators or responsiveness to treatment in persons with cLBP. We further evaluated practicality and feasibility, considering factors such as length of questionnaires, conceptual clarity, and simplicity of items and scoring. The BWG aimed to include both resilience factors as well as risk factors and aimed to balance breadth of conceptual domain coverage with brevity of validated questionnaires in order to minimize participant burden. Final decisions regarding recommendations of specific questionnaires were made by consensus.

Recognizing that BACPAC projects differ regarding their aims and interests, the BWG also reviewed discretionary or optional domains and questionnaires. While BWG recommended questionnaires are meant to supplement the HEAL CDE and BMD for all BACPAC projects, the discretionary questionnaires are options that can be administered in studies where the additional participant burden is not a barrier, and where the concept is particularly relevant to the study aims.

The BWG QST subgroup developed best practice guidelines for harmonizing psychophysical testing across BACPAC sites conducting QST. The recommendations of the BWG were documented and provided to the BACPAC Executive and Steering Committees, reviewed, and revised as needed until approval was received from the Steering Committee. The members of this subgroup searched the literature for studies that used QST procedures in cLBP, compiled a list of the most commonly used QST procedures in this patient population, and developed a minimal set of QST procedures through a consensus process.

The primary work of the BWG was accomplished during January to May 2020. However, members of the BWG have reconvened as needed to address additional issues as they would come up, including modifications to procedures and additional questions needed as the BACPAC projects readied for recruitment of participants.

## Results and Recommendations

### Patient-reported Outcomes/Psychosocial Domains and Questionnaires

**Figure 1.**
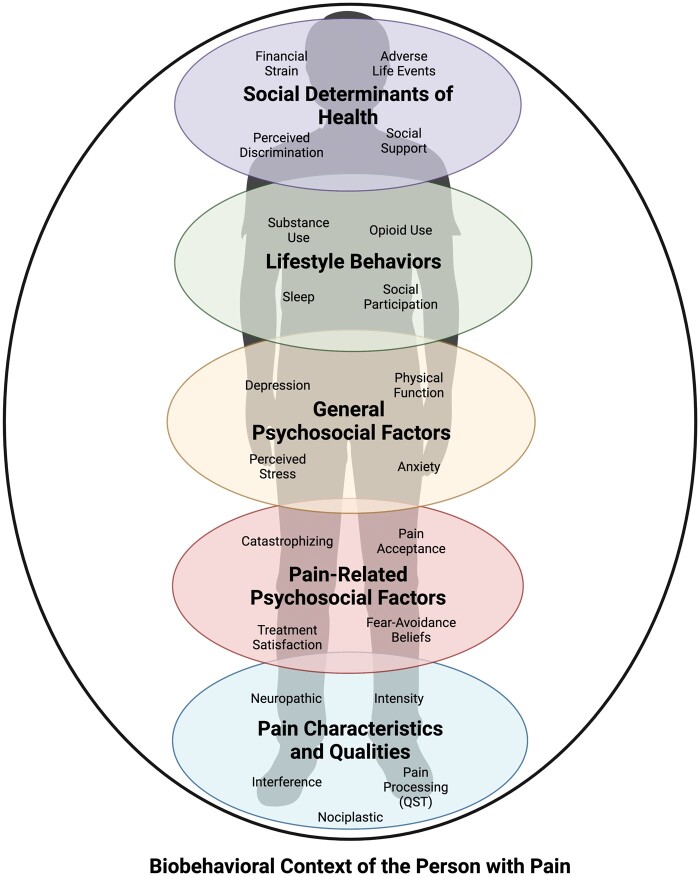
Biobehavioral, psychosocial and social context domains relevant to persons with chronic pain, with key categories and constructs.

BWG-recommended PRO/psychosocial domains for inclusion in BACPAC are pain characteristics, pain-related psychosocial and behavioral factors, general psychosocial factors, lifestyle choice factors, and social determinants of health (SDoH)/social factors (Figure 1). Each of these domains include several categories or constructs. In the following paragraphs we summarize the domains, domain constructs, and list specific recommended and discretionary/optional questionnaires. Table 2 lists the domains, constructs, and questionnaires, and identifies the BWG-recommended questionnaires that are also included in the NIH HEAL CDE (required in HEAL studies in humans) and the BMD (required in BACPAC studies). As indicated in Table 2, the BWG added several recommendations and options for broad inclusion beyond the BMD and HEAL CDE. BWG-recommended questionnaires are intended for use in all BACPAC projects. The discretionary questionnaires listed on Table 2 are validated but non-required options that may be chosen based on the aims and interests of particular BACPAC projects.

**Table 2:** PRO/Psychosocial domains and patient-reported questionnaires

DOMAIN Construct	BWG RECOMMENDED (REC) and DISCRETIONARY (DISC) Patient-Reported Questionnaires (Number of Items)	REC, DISC	BACPAC Minimum Dataset (BMD)	NIH HEAL Common Data Element (CDE)
Pain Characteristics and Qualities				
Pain intensity	PEG (3)	REC	X	X
	Pain Intensity NRS for low back pain (1)		X	
Pain interference	PEG (has both pain intensity and interference) (3)	REC	X	X
	PROMIS-29 + 2 Pain Interference (4)		X	
Pain duration and frequency	Low-back pain duration and frequency (items from NIH RTF MDS) (2)	REC	X	
Pain location spread	Radicular pain questions adapted from NIH RTF MDS (2)	REC	X	
Pain somatization	Bothersome stomach pain, headaches (2)	REC	X	X
Nociplastic pain (CNS sensitivity)	Widespread Pain Inventory (7)	REC	X	
	Michigan Body Map (1)	REC		
Neuropathic pain	PainDETECT (9)	REC		
Sensory vs Affective pain	McGill SF (16)	DISC		
Pain-related disability	Oswestry Disability Index (10)	DISC		
Pain-related Psychosocial				
Pain catastrophizing	PCS SF (6)	REC	X	X
Global Satisfaction with treatment	Patient Global Impression of Change (since start of a treatment) (1)	REC	X	X
Fear of movement	FABQ-Physical Activity scale (5)	REC		
	Tampa Scale of Kinesiophobia (17)	DISC		
Pain self-efficacy	PSEQ (4)	REC		
Pain acceptance	CPAQ (8)	REC		
Expectations of improvement	Expectation of pain relief (with or without treatment) (1)	DISC		
	HEAL Treatment Expectancy item (1)	DISC		
Pain avoidance	Pain Anxiety Symptoms Scale SF (20) Avoidance and Physiological Pain Anxiety subscales	DISC		
Pain coping strategies	Coping Strategies Questionnaire-brief version (24)	DISC		
Psychosocial factors—General				
Physical function	PROMIS Physical Function—6b (6)	REC	X	X
Depression	PHQ2 Depression (2)	REC	X	X
	PROMIS-29 + 2 Depression (4)	REC	X	
Anxiety	GAD2 Anxiety (2)	REC	X	X
	PROMIS-29 + 2 Anxiety (4)	REC	X	
Stress	PSS (4, or 10)	REC		
Affect	PANAS SF (10) or Positive affect alone (5)	DISC		
Optimistic outlook	HEAL Positive outlook scale (6)	DISC		
Cognition	PROMIS-29 + 2 memory and concentration (2)	DISC		
Interoceptive awareness	MAIA-2 or selected subscales (37)	DISC		
Self-efficacy	PROMIS Self-efficacy for Managing symptoms (4), or PROMIS General Self-efficacy (4)	DISC, DISC		
Lifestyle behaviors				
Sleep	PROMIS Sleep Disturbance 6a (6)	REC	X	X
	Sleep duration (hours, minutes per night) (1)	REC	X	X
	PROMIS Sleep-Related Impairment 4a or 8a (4, 8)	DISC		
Substance use	TAPS Screener—part 1 (5)	REC	X	X
Opioid medication	Current daily opioid use (1)	REC	X	X
Social participation	PROMIS-29 + 2 ability to participate in social roles and activities (4)	REC		
Social Determinants of Health (SDOH)				
Adverse life events	PC-PTSD-5 Exposure to traumatic event(s) (1–7) adapted to include childhood or adulthood exposure question (1), or	DISC		
	Life Events Checklist (17)	DISC		
Financial strain	Difficulty paying for basic needs (1)	DISC		
	Thrive SDOH tool (11)	DISC		
Perceived discrimination	Perceptions of being treated unfairly due to 1) race, ethnicity, and 2) sexual orientation or gender identity (1 or 2 questions)	DISC		
Social support	PROMIS Emotional Support 4a v2 (4)	DISC		
	MOS Social Support (includes emotional and instrumental support) (19)	DISC		

CNS = Central Nervous System; CPAQ = Chronic Pain Acceptance Questionnaire; GAD2 = Generalized Anxiety Disorder screener, 2 item version; FABQ = Fear Avoidance Beliefs Questionnaire; HEAL = Healing Encounters and Attitudes Lists; MAIA-2 = Multidimensional Assessment of Interoceptive Awareness-2; MOS = Medical Outcomes Study; NIH RTF MDS = National Institutes of Health Research Task Force Minimum Data Set; NRS = Numeric Rating Scale; PANAS = Positive and Negative Affect Schedule; PC-PTSD-5 = Primary Care Post-Traumatic Stress Disorder screener for DSM5; PCS = Pain Catastrophizing Scale; PEG = Pain, Enjoyment, General activity; PHQ2 = Patient Health Questionnaire depression screener, 2 item version; PROMIS = Patient Reported Outcomes Measurement Information System; PSEQ = Pain Self-Efficacy Questionnaire; SF = Short Form; TAPS = Tobacco use, Alcohol use, Prescription medication misuse, illicit Substance use.

The PROs/psychosocial questionnaires subgroup aimed for broad coverage of domains and categories relevant to chronic pain and health outcomes, while at the same time choosing validated questionnaires for which concise and precise versions, or “short forms” were available. For example, the PROMIS-29 + 2 health profile [18–25] was recommended because it includes brief (1–4 item) measures of 8 health-related areas: Physical Function, Pain Interference, Pain Intensity, Sleep, Depression, Anxiety, Fatigue, Ability to Participate in Social Roles and Activities, and Cognitive Function. Notably, comprehensive conceptual coverage of all potentially relevant categories was not intended. For instance, patient-reported physical activity is important to measure in cLBP studies [8, 26]. However, an optimally brief physical activity questionnaire for use across BACPAC sites was not identified. Rather, several BACPAC sites assess physical activity objectively via at-home monitoring (described in the BACPAC Biomechanical and Physical Function WG manuscript) and may also administer a patient-report measure of physical activity of their choice.

#### Domains, Constructs, and Questionnaires

Pain Characteristics and Qualities. Pain intensity and interference with life activities are assessed with the three-item PEG (Pain, Enjoyment, General Activity) [27] and with PROMIS-29 + 2 Pain Intensity and Pain Interference [28]. The duration and frequency of low back pain are assessed directly via two BMD questions. Radicular pain (spread of back pain to the legs) and somatization (bothersome headaches and stomach pain) are assessed via four BMD questions. The Widespread Pain Index [29] and the Michigan Body Map [30] assess additional pain locations and are helpful clues to the presence of nociplastic pain or central nervous system sensitization. For assessing whether pain is of neuropathic origin, we recommend the PainDETECT questionnaire [31]. For projects interested in assessing sensory versus affective pain, the Short-form McGill Pain Questionnaire [32] is a good option. Pain-related disability can be assessed using the Oswestry Disability Index (ODI) [33].

Psychosocial Factors—Pain-Related. Ways of thinking about pain and strategies to cope with pain have an impact on patients’ behavior and quality of life. Pain catastrophizing, or ruminating about and magnifying pain, and having a sense of helplessness, is assessed by the BMD-required Pain Catastrophizing Scale, six-item version [34–36]. The BWG recommends that fear of movement be assessed by the physical activity subscale of the Fear Avoidance Beliefs Questionnaire (FABQ) [37]; however, the Tampa Scale of Kinesiophobia [38, 39] is an alternative option. A similar concept is pain avoidance, which can be assessed via the Pain Anxiety Symptoms Scale short form [40, 41] at a BACPAC project’s discretion. Adaptive coping with pain is also important to assess. Pain acceptance is measured via the Chronic Pain Acceptance Questionnaire-8 (CPAQ) [42, 43]. Pain self-efficacy, or one’s belief in being able to effectively control or cope with pain, is a BWG recommended construct that can be assessed via the four-item version of the Pain Self-Efficacy Questionnaire [36]. If a BACPAC project is particularly interested in assessing a broad range of Pain coping strategies (PCS), the 24-item version of the Coping Strategies Questionnaire [44, 45] is an option. As Pain Catastrophizing is one of the subscales, this subscale can be dropped to avoid redundance with the PCS. For BACPAC projects that assess responses to pain treatment, global satisfaction with treatment is important, and the BMD requires the single-item Patient Global Impression of Change [46] on which patients can rate either the improvement or worsening of symptoms on a seven-point scale. Expectations of improvement is an optional category that can be rated with a single-item expectation of pain relief over a 6-month period, or a single Treatment Expectancy item from the Healing Encounters and Attitudes Lists [47]. There are many other well-validated questionnaires that have significant conceptual overlap with the questionnaires listed above.

Psychosocial Factors—General. Self-report of physical function is assessed via the PROMIS Physical Function 6 b short form, as required by the BMD. This questionnaire adds two items to the PROMIS-29 + 2 health profile, which includes four physical function items. Depression and anxiety are assessed with two-item CDE screening questionnaires, the Patient Health Questionnaire-2 (PHQ2) [48, 49] and Generalized Anxiety Disorder-2 (GAD2) [50]. The BMD added the PROMIS-29 + 2 Depression and Anxiety scales [19], which each includes four items. The BWG recommends assessing stress with the Perceived Stress Scale [51], which has 10-item and 4-item versions. Several discretionary or optional categories include affect, measured by the Positive and Negative Affect Schedule [52, 53], Optimistic attitude (Positive Outlook short form of the Healing Encounters and Attitudes Lists) [47], and general self-efficacy, for which PROMIS has several scales, such as general self-efficacy and self-efficacy for managing symptoms [25]. Cognition can be briefly assessed with 2 items about Memory and Concentration ability from the PROMIS-29 + 2 [19]. Interoceptive awareness, which measures various beliefs and behaviors regarding bodily sensations, including pain, can be assessed optionally using the Multidimensional Assessment of Interoceptive Awareness, version 2 (MAIA-2) [54]. This questionnaire, however, is lengthy and allows for selecting individual scales (e.g., for assessing different habitual attention styles towards pain, including ignoring pain).

Lifestyle Behaviors. Sleep is a BMD-required category and is assessed via the PROMIS Sleep Disturbance 6a short form [55], of which four of the six items are included on the PROMIS-29 + 2 profile, and patient-report of hours and minutes of sleep, on average [56]. PROMIS Sleep-related Impairment 4a or 8a is an additional option. Substance use, assessed with the Tobacco, Alcohol, Prescription Medication (TAPS) [57] screener part 1 and opioid medication (current daily dose) are BMD and CDE requirements. Social participation is an additional recommendation of the BWG and is assessed with the four-item PROMIS Ability to Participate in Social Roles and Activities short form, which is included within the PROMIS-29 + 2. The BWG PROs/psychosocial factors subgroup did not make a specific recommendation for a perceived physical activity questionnaire.

Social Determinants of Health (SDoH). BACPAC projects, particularly those engaging in cLBP phenotyping, may include questions to assess social determinants of health. Adverse life events may be assessed via the Primary Care-Post Traumatic Stress Disorder (PC-PTSD-5) [58] five-item screening instrument or with longer questionnaires such the Life Events Checklist [59]. Financial strain may be assessed with a single item adapted for use in BACPAC MRCs that asks about difficulty paying for basic needs such as food, medical care, and utilities [60]. Alternatively, the THRIVE SDoH tool [61] may be used. Perceived discrimination based upon race, ethnicity, or color, and sexual orientation or gender identity can be included. The BWG developed 1–2 perceived discrimination questions in consultation with the Program Director at the National Institute on Minority Health and Health Disparities (NIMHD). Perceptions of social support can be assessed with PROMIS Emotional Support 4a v2 or the Medical Outcomes Study (MOS) Social Support questionnaire [62], which includes subscales of emotional and instrumental support.

Other categories discussed by the PROs/psychosocial questionnaires subgroup of the BWG and that may be of interest to BACPAC or other investigators included: Mindfulness (Five Facet Mindfulness Questionnaire; 63], Pain Behavior (PROMIS Pain Behavior), Personality (60-item NEO short form; 64], and Emotion Regulation.

#### QST Working Subgroup Recommendations

The QST Subgroup recommended a core set of two QST procedures that would be performed—at minimum—at all BACPAC sites conducting QST (Table 3). These include assessment of pressure pain threshold (PPT) and temporal summation at the lumbar region (primary pain site) and at a remote region as a control site. These QST assessments have demonstrated good to excellent reliability in cLBP [65], neuropathic pain [66, 67], and non-pain samples [68]. There was an understanding that some sites may choose to include additional QST procedures such as conditioned pain modulation (CPM). It was agreed that the order in which these procedures were performed was important and should be standardized across sites. The core set of QST tests are listed below in the recommended order that they should be performed, prior to any additional site-specific QST procedures and with testing of the control site always preceding that of the painful lumbar region.

**Table 3.** Minimum set of QST procedures

QST Procedure	Description	Measurement
Pain Pressure Threshold (Algometry)	An algometer with a 1-cm^2^ rubber probe is applied at a rate of 0.5 kgf/second until the participant first reports that the pressure sensation becomes painful. The probe is applied over the primary pain site and the contralateral trapezius as a control site.	Average of three trials at each body site, recorded as pressure intensity in kgf/cm^2^
Temporal Summation (multiple pinpricks)	Neuropen device with a 40 g Neurotip is used to apply a train of 10 identical pinpricks at a rate of 1 Hz over the primary pain site and the volar forearm as a control site. Participant is asked to rate pain intensity of the 1^st^ and 10^th^ pinprick, and 15- and 30-seconds after the train of pinpricks.	Average of three trials at each body site, recorded as the difference between the 1st and 10th pinprick sensation, and average of pain aftersensation ratings.

- *Pressure pain threshold* is assessed using a hand-held algometer with a 1-cm^2^ rubber probe (FPK20 or FPX25, Wagner Instruments, Greenwich, CT, USA). The primary test site is located in the lumbar region by participants’ identification of their most painful site in response to manual over-pressure (springing palpation) performed in the prone position. The control site is located over the contralateral upper trapezius muscle (diagonal from lumbar site). Pressure is manually increased at a rate of rise of 0.5 kgf/cm^2^/s (10 kgf/cm^2^ max, metronome guided) until participants first report that the pressure sensation becomes painful. Pressure intensity (in kgf/cm^2^) read from the algometer at that time is considered the PPT. Measurements are conducted 3×/site with 60-second rest intervals between each pressure application. Probe placement is varied slightly trial to trial to prevent sensitization from repeated testing of the same site. Mean PPT of the three trials is used for analysis.
- *Temporal summation* measures increases in excitatory pain pathways and is thought to reflect the progressive increase in dorsal horn neuronal firing in response to repetitive C-fiber stimulation [69–73]. Enhanced temporal summation is common in chronic pain, including in subsets of patients with cLBP, and is predictive of pain and treatments outcomes [74, 75]. A Neuropen device with a 40-gram Neurotip (Owen Mumford, Oxfordshire, United Kingdom) is used to apply a series of three sets of 10 identical pinprick stimuli applied at the rate of 1 Hz (metronome guided) to both control and primary pain sites. The dominant volar forearm serves as the non-painful control site. The primary pain site is the painful area in the lumbar region, as previously identified in the assessment of PPT done previously. Following each train of 10 stimuli, participants are asked to rate the magnitude of pain sensations of the 1st and 10th pinprick using a 0–10 numerical rating scale (NRS; 0 = no pain, 10 = worst imaginable pain). Temporal summation for each site is calculated as the mean difference in pain ratings evoked by the 1st and 10th stimuli. Participants also rate any ongoing *pain aftersensations* at 15- and 30-second following each train of stimuli.

Optional—Conditioned Pain Modulation. There is significant controversy in literature over the optimal procedures to elicit a robust CPM, as well as the optimal CPM techniques for predicting responses to treatment and using CPM as a pain treatment biomarker. One of the key scientific gaps that is necessary to fill is the testing of multiple CPM techniques in large populations of patients with chronic pain and relating the CPM findings to treatment outcomes. Therefore, the BWG recommended that the most harmonious approach is to have different sites in BACPAC implement different approaches to measure CPM, with the idea of being able to compare and contrast results from these methods at the study’s conclusion.

*Example of CPM with pressure pain as the test stimulus and cold water as the conditioning stimulus.* CPM procedures require a conditioning stimulus to induce endogenous analgesic systems and alter pain perception, and a test-stimulus to evaluate the endogenous analgesic response to the conditioning stimulus. CPM is attenuated in the majority of chronic pain participants and its magnitude is predictive of a variety of pain outcomes [76, 77]. Here, immersion of one hand into a circulating cold water bath (4–12°C; NESLAB Digital One RTE 7, Thermo Scientific, Newington, NH, USA, or similar) will serve as the conditioning-stimulus and PPT at the contralateral trapezius will serve as the test-stimulus. This method is consistent with that of Locke [78] and others [79, 80]. Baseline measurement of the test-stimulus will be acquired during the assessment of pressure pain threshold. Conditioning stimulation will begin by immersing the hand to a level 10 cm above the wrist into the water bath. The hand will be immersed for a total of 60–90 seconds of hand immersion; trapezius PPT will be re-measured 1–2 times while the hand is still immersed in the cold water. CPM magnitude will be calculated as the difference in mean PPT measured prior to and during the conditioning stimulus, with increases in PPT during conditioning interpreted as evidence of efficient endogenous pain inhibition.

## Discussion

The BWG identified key PRO/psychosocial domains for harmonization and identified well-validated questionnaires to represent the domains. For various reasons specific to the science of each project, not every BACPAC project will include all the same questionnaires. Most of the questionnaires discussed in this paper are required because they are included in the BMD and/or the NIH HEAL CDE. The BWG added several recommended questionnaires and discretionary or optional questionnaires beyond the BMD and HEAL CDE. The BWG psychosocial questionnaires subgroup acknowledges that there is overlap among domains and within the conceptual areas measured by individual questionnaires. We also acknowledge that various professions may have different views about which domains are most important to assess.

One limitation of the work reported here is that the recommendations of the BWG were not based upon an exhaustive systematic review of all possible domains and all existing psychosocial and psychophysical measures that may be relevant to cLBP. Furthermore, the BWG did not comprehensively review all domains and questionnaires that may be important in phenotyping cLBP. For example, the BWG did not identify a questionnaire to assess patient reports of their own physical activity. Furthermore, we did not include all possible domains of SDoH that are assessed in clinical settings but have not yet been validated in research settings. The assessment of psychosocial and behavioral factors affecting pain and influenced by pain could be endless. Likewise, QST batteries can be lengthy and time consuming. It was important to recommend brief assessments where possible to minimize participant burden.
